# Dynamics of novel forests of *Castilla elastica* in Puerto Rico: from species to ecosystems

**DOI:** 10.1002/ece3.1578

**Published:** 2015-07-22

**Authors:** Jéssica Fonseca da Silva

**Affiliations:** 1 International Institute of Tropical Forestry, USDA Forest Service1201 Calle Ceiba, Jardín Botánico Sur, Río Piedras, 00926-1119, Puerto Rico; 2 Center for Applied Tropical Ecology and Conservation, University of Puerto RicoFacundo Bueso Building, Office 301-A, San Juan, 00931, Puerto Rico; 3 Department of Biology, University of Puerto Rico - Río Piedras CampuPO Box 23360, San Juan, 00931-3360, Puerto Rico

**Keywords:** Biomass increment, introduced species, litter dynamics, survival rate, tree growth

## Abstract

Novel forests (NFs)—forests that contain a combination of introduced and native species—are a consequence of intense anthropogenic disturbances and the natural resilience of disturbed ecosystems. The extent to which NFs have similar forest function as comparable native secondary forests is a matter of debate in the scientific community. Little is known about the performance of individual species in those forests. This study focuses on the functional attributes of *Castilla elastica* NFs in Puerto Rico and on the differences between introduced and native species growing side by side in these forests. Rates of processes measured here were later compared with data from literature about NSFs. I hypothesize that juvenile plants of *C. elastica* in NFs have higher survival rate than those of native species and that *C. elastica* trees have faster biomass fluxes than native trees. To test the hypotheses, I measured survival rates of juvenile plants and tree growth and characterized the aboveground litter fluxes and storage. Although juvenile plants of native species displayed higher survival rates than those of *C. elastica* (53% vs. 28%), the latter was dominant in the understory (96%). Stand biomass growth rate was 2.0 ± 0.4 (average ± one standard deviation) Mg·ha^−1^·year^−1^ for the whole forest, and *Guarea guidonia*, a native species, exhibited the highest tree growth. Total litter fall was 9.6 ± 0.5 Mg·ha^−1^·year^−1^, and mean litter standing stock was 4.4 ± 0.1 Mg·ha^−1^. *Castilla elastica* litter fall decomposed twice as fast as that of native species (5.8 ± 1.1 vs. 3.03 ± 1 *k*·year^−1^). Literature comparisons show that the present NFs differ in some rates of processes from NSFs. This study brings unique and detailed supporting data about the ecological dynamics under mature novel forest stands. Further comprehensive studies about NFs are important to strengthen the body of knowledge about the wide range of variation of emerging tropical ecosystems. Due to the large increase in the area covered by NFs, greater attention is needed to understand their functioning, delivery of ecological services and management requirements.

## Introduction

Human populations have caused dramatic changes to the composition of forest communities by altering biological and physical attributes of ecosystems. Native populations can be displaced when they are no longer able to cope with disturbances. Thus, modified ecosystems experience increased rates of species losses, species introductions (Noble 1989; Hooper et al. 2005), and continuous rearrangement of the community (Tilman 1987; Chapin et al. 1997; Lugo 2009; Hobbs et al. 2013). New communities, under the influence of introduced species, can bring functional changes to ecosystems, such as altered productivity and carbon flows (Chapin et al. 2000; Loreau et al. 2001; Hooper et al. 2005).

Many naturalized species grow faster than native species and establish successfully into disturbed environments. Common traits often advantageous for species establishment are faster growth rate (Oba et al. 2001; Grotkopp et al. 2002; Caño et al. 2008), higher phenotypic plasticity (DeWalt et al. 2004; Caño et al. 2008), and higher seedling survival rates (Grotkopp et al. 2002). Moreover, stands dominated by introduced species show high primary productivity and litter fall (Gordon 1998; Wilsey and Polley 2006; Vilà et al. 2011). Nevertheless, introduced species are mostly successful in areas disturbed by human activities where native species are unable to grow (Goodenough 2010; Lugo et al. 2012).

Over the past few decades, some tropical countries have experienced increasing natural forest regeneration on abandoned cultivated lands (Hobbs et al. 2006; Chazdon et al. 2009). Emerging ecosystems, called novel forests (NFs), are a consequence of these land abandonment processes (Mascaro et al. 2008; Hobbs et al. 2009; Hobbs 2012; Lugo et al. 2012). These forests are known as novel because they contain species combinations that are new to the landscape (Milton 2003; Lugo and Helmer 2004). By now, about 30% percent of the total world's surface area is already covered by novel ecosystems (Perring and Ellis 2013), whose characteristics have been recently reviewed (Lugo et al. 2012; Hobbs et al. 2013).

Emerging NFs have been a subject of controversy, in particular because of uncertainty of to whether these forests have similar function as native secondary forests (NSFs)— regenerating forests under dominance of native species. Despite that, some studies have already shown that NFs can have similar ecosystem attributes to NSFs, such as providing refuge for native biodiversity, maintenance of the water cycle, and productivity (Abelleira-Martínez 2010, 2011; Mascaro et al. 2012; Pethiyagoda 2012). The study of the functioning and species performances in the same forest can help us understand conservation roles. Considering the biodiversity found in NFs and the large area they occupy, further research is imperative to understand not only their functioning but also to identify specific management strategies.

**Figure 1:**
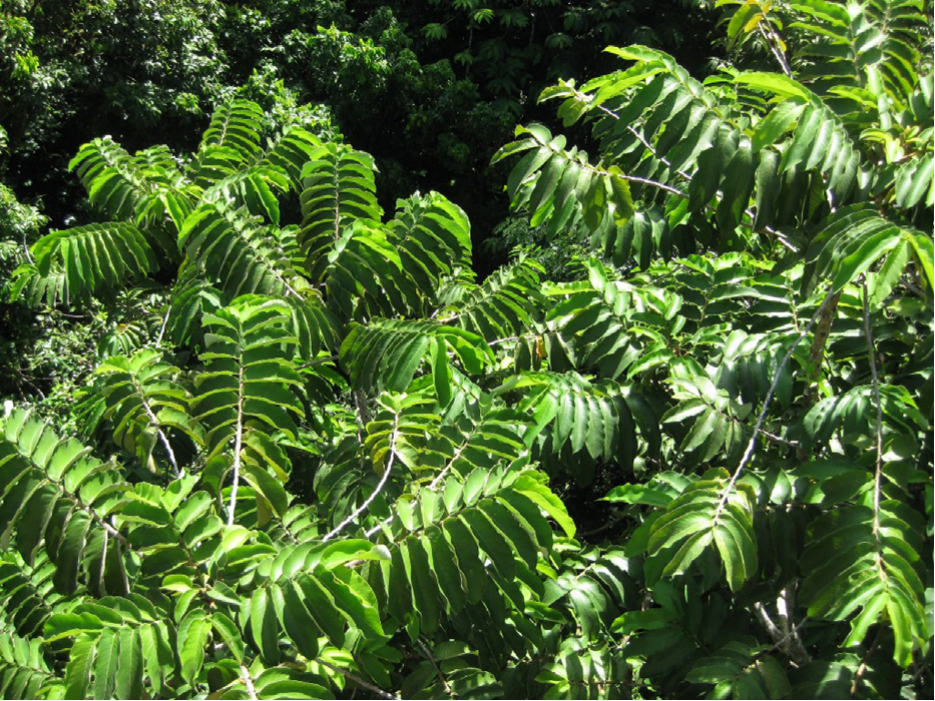
Canopy of *Castilla elastica* novel forest seen from above, highlighting *C. elastica* trees.

Puerto Rico, in the Caribbean Sea, has experienced substantial growth (Aide et al. 1996; Zimmerman et al. 2000; Lugo 2009) as a result of natural regeneration that followed previous intensive disturbances (Picó 1969) and species introductions (Lugo 2009). One example are the forests of *Castilla elastica* Sessé (Moraceae [Fig. 1]) that have been regenerating for more than five decades and offer ideal opportunities for studying the ecological attributes of novel ecosystems.

This study aims to compare the ecology of *C. elastica* trees with native trees growing side by side in *C. elastica* NFs. Additionally, I use the present results to compare the functioning of NFs with NSFs reported in the literature. I hypothesize that juvenile plants of *C. elastica* show higher survival rate than those of native species and that *C. elastica* trees have faster tree growth and litter fluxes. I also expect faster rates of stand-level growth and biomass fluxes for the Castilla stands over NSFs. To test the hypotheses, I measure the survival rates of juvenile plants, tree growth, and aboveground litter dynamics in two stands of *C. elastica* NFs.

## Materials and Methods

### Study area

This study was conducted in the humid karst region of Puerto Rico at the biological reserve El Tallonal (18°24′27″N 66°43′53″W). The soil type is the *Almirante arcilloso* (humid Oxisol [Beinroth et al. 2003; Viera-Martínez et al. 2008]) and the vegetation grows in a subtropical moist forest zone (Holdridge 1967; Ewel and Whitmore 1973), with average temperature and precipitation of 25.5°C and 1295 mm, respectively. The dry season lasts from January to April and the rainy season from July to September.

Agriculture and cattle grazing were common in the study area until the 1950's. Forest regeneration occurred naturally after land abandonment. The Importance Value Index (IVI) that considers tree density, frequency and total basal area was 37% for *C. elastica* in the study sites (Fonseca-da Silva 2014). Historical analyses from aerial photographs (taken in 1936, 1963, 1977, 1983, 1995, and 2005) show that stands where *C. elastica* is dominant have been regrowing for more than 40 years. Besides *C. elastica* trees with high IVI include *Guarea guidonia* (L.) Sleumer (14%) and *Cecropia schreberiana* Miq. (9%), both native species.

I chose two replicated stands (mentioned here as study sites) with the following geolocations, estimated age (in 2005), area (in hectares), and elevation (meters above sea level [masl]):

Tallonal 1 = 18°24′23.84″N, 66°44′05.25″W, 50 years, 0.9 ha, and 122 masl;
Tallonal 2 = 18°24′30.64″N, 66°43′45.85″W, 40 years, 0.5 ha, and 116 masl.

### Sampling and processing

I measured air temperature, relative humidity, photosynthetically active radiation, and soil water content using specific onset HOBO sensors coupled with a portable data logger (Appendix 1). Here I considered juvenile plants as young trees <1 m in height, including recently recruited seedlings and older saplings. I measured the survival rate in 12 subplots of one m^2^, randomly placed across the study sites. A total of five subplots were established at each study site. Additionally, two subplots were placed in a third stand at El Tallonal, at which I only measured juvenile plants dynamics. This gives a total of 12 subplots in the area of study. Care was taken to avoid forest edges and big gaps. I counted, tagged, and identified every individual to genus or species level when possible. Subplots were re-surveyed after eight months to assess the survivorship.

I used the point quarter method (Cottam and Curtis 1956) to measure the vegetation structure underlying the biomass calculations. Two perpendicular 100-m transects, with ten randomly chosen points each, were established at each study site where I measured 80 trees with ≤2.5 cm ≥ 10 cm DBH (small trees) and 80 trees with ≥10 cm DBH (large trees). At each point, I selected the four large and small trees closest to the centre, for a total of 80 trees per transect and 160 trees per study site (Fonseca-da Silva 2014). I measured the diameter at breast height (DBH) at 1.37 m in height and identified trees using taxonomic guides for Puerto Rico (Little et al. 2001 and the PLANTS web database: http://plants.usda.gov/java/). Because many trees had multiple stems, I reported growth as individual stem growth (in basal area and in biomass). Stems were measured three times to obtain their net DBH increment: The first censuses were in 2007 at Tallonal 1 and in 2008 at Tallonal 2; both sites were measured again in June 2011. Care was taken to measure the DBH in the same place as the previous census, that is, one cm below the tag. Additionally, ten wood cores were collected from large and small trees (chosen randomly) using a 2.94-cm^3^ cylinder. Wood cores were dried to constant weight at 105°C, and then weighed to obtain the specific wood density (g·cm^−3^). I used literature data for those species for which I lacked the wood density information (Francis 1995, 2000; Wittmann et al. 2008).

A total of 20 litter baskets (ten baskets per study site) measuring 0.25 m^2^ and 50 cm in height were installed and emptied biweekly from May 2008 to May 2009. Baskets were distributed on alternate sides along a 100-m transect in each study site. A 0.25-m^2^ frame was used to collect ground litter samples (ten monthly), following the litter baskets distribution; that is, a random sample of ground litter was collected near each basket. Care was taken to avoid sampling the same spot more than once.

Litter samples were oven dried for three days at 65°C to constant weight, and then sorted and weighed separately. *Castilla elastica* leaves, flowers and fruits were sorted from those of native species to assess their respective fluxes and stocks. I used the following categories for sorting litter fall: *C. elastica* leaves, native leaves, flowers plus fruits of *C. elastica*, flowers plus fruits of native species, wood, and miscellaneous vegetation fragments. Samples of litter standing stock were sorted in the same categories cited above, as well as detritus, which included soil particles and vegetation fragments smaller than 0.20 mm.

### Data analyses

I expressed survival rates of juvenile plants as the relative frequency (percent) of the total individuals counted in the first census. Demography was also reported using the species percentage of total counted individuals (Appendix 2).

I calculated the basal area and tree density according to Cottam and Curtis (1956) for both time 1 and time 2 censuses (*T*_1_, first time surveyed; *T*_2_, last time surveyed). Basal area increment and stem growth (cm^2^·year^−1^) were calculated from the DBH net increase from *T*_1_ to *T*_2_, and compared among species. Weighed wood densities were estimated for each study site based on the species IVI. Tree height, used later for growth calculations, was estimated using a clinometer (*n* = 6 and *n* = 10, for large and small trees, respectively).

Tree height (H, cm), weighed wood density (WD, g·cm^−3^), and basal area net increment (BA_n_, cm^2^·year^−1^) were used to calculate biomass growth (G, g·year^−1^) for each species as follows: G = H * WD * BA_n_. Stand-level growth was then calculated using the average biomass growth (G) per site, multiplied by the stand tree density reported in the literature for the same sites (Appendix 3).

Total litter fall and mean litter standing stock were used to estimate a turnover rate constant (*k*·year^−1^) and residence time (1/*k*) as described by Olson (1963). To justify these calculations, I previously verified that litter fall dynamics was in a steady state, which means that the amount of litter fall and stock are in balance after a year. Additionally, I used values of total monthly litter fall minus the difference between the stocks of the previous and the current months as another method for estimating decomposition (Lugo 1992).

Statistical analyses were made using JMP version 8.0.1 (SAS Institute Inc., Cary, NC), and the level of significance for the analyses was set at *P* ≤ 0.05. Data were previously checked for normality before performing any of the following statistical tests. To check whether introduced species have greater survival rates, I tested the mean survivorship of grouped native vs. introduced species (mostly *C. elastica*) using a *T*-test. I tested the difference in stem growth between *C. elastica* and *G. guidonia* using a *T*-test. Data variability of litter fall and litter standing stock throughout the year was tested with ANOVA. Monthly litter fall, leaf fall, and flowers plus fruits of *C. elastica* were compared with native species respective categories using a *T*-test.

### Literature search

Original research articles and reviews about forest dynamics were selected for comparison. Originally, I chose native secondary forests and novel forests that were comparable with the Castilla NFs, restricting to tropical and subtropical moist forests (*sensu* Holdridge 1967) and focusing mainly on those growing on karst substrate with similar age. However, few studies reported data about the same forest attributes measured here. To address this problem I included forests growing on other substrates for broader comparisons.

## Results

### Survivorship of juvenile plants

The relative survival rate of *C. elastica* was lower than that of grouped native species (28 ± 0.04 and 53 ± 0.1 percent, respectively [*t* = 2.1, df = 11.8, *P* < 0.03]). However, juvenile plants of *C. elastica* accounted for 96.3% of total 1452 individuals, while combined individuals of native species represented only 2.1% of total individuals found in the subplots (Appendix 2). Other introduced species, *Coffea arabica* L. and *Syzygium jambos* L. Alston, accounted for 0.3% of total individuals. The last 0.8% could not be identified to species level.

### Stem growth and stand biomass increment

Stems showed a mean growth rate in basal area of 6 ± 1.2 cm^2^·year^−1^ (*n* = 342 [Table 1]). Nineteen percent of total stems reduced slightly in circumference, 45% grew <1 cm^2^·year^−1^, and 36% of stems grew up to 166 cm^2^·year^−1^. *Guarea guidonia* grew on average 23.6 ± 2.5 cm^2^·year^−1^, and it was the only native species that significantly differed from rates of *C. elastica* (4.5 ± 3.6 cm^2^·year^−1^) (*t* = −2.6, df = 33, *P* ≤ 0.05). Weighed wood densities for all trees and each study site were, respectively: 0.37, 0.29, and 0.33 g·cm^−3^ (Appendix 3). Stand biomass growth was 2.0 ± 0.4 Mg·ha^−1^·year^−1^ (Table 1).

**Table 1:** Mean stem growth ± one standard error (number of observations) in novel forests of *Castilla elastica*. Stand-level biomass growth was calculated using wood density, tree height, and tree density, for large (≥10 cm of diameter at breast height [DBH]), small (≥2.5 < 10 cm of DBH), and all stems (≥2.5 cm of DBH)

Stem growth in basal area (cm^2^·year^−1^)			
Study site	Large	Small	All
Tallonal 1	11.6 ± 3.8 (80)	0.4 ± 0.1 (86)	5.8 ± 1.9 (166)
Tallonal 2	10.8 ± 2.9 (95)	0.3 ± 0.1 (74)	7.5 ± 1.6 (169)
Mean	11.2 ± 2.3 (176)	0.36 ± 0.1 (161)	6.0 ± 1.2 (336)

Stand-level growth (Mg·ha^−1^·year^−1^)			
Place	Large	Small	All
Tallonal 1	4.3 ± 1.4 (80)	0.16 ± 0.06 (86)	2.1 ± 0.7 (166)
Tallonal 2	3.3 ± 0.8 (95)	0.05 ± 0.02 (74)	2.2 ± 0.5 (170)
Mean	3.7 ± 0.8 (175)	0.12 ± 0.03 (160)	2.0 ± 0.4 (336)

### Aboveground litter dynamics

#### Litter fall

**Figure 2:**
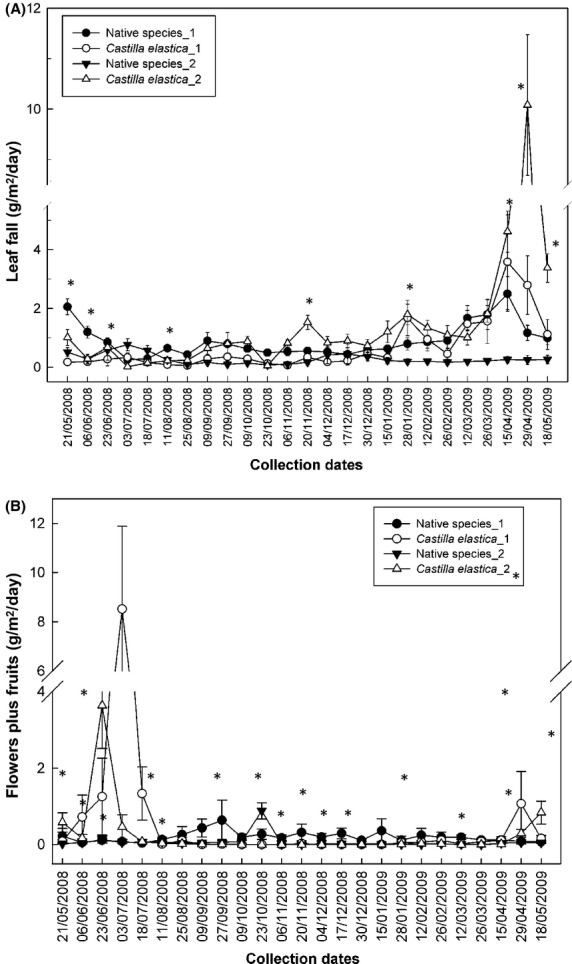
(A) Leaf fall of *Castilla elastica* and grouped native species for each study site 1 = Tallonal 1; 2 = Tallonal 2. (B) Necromass of flowers plus fruits. Numbers indicate study sites identity. (*) = *P* ≤ 0.05 among species.

Total leaf fall was higher for *C. elastica* than for native species (3.9 ± 0.3 vs. 2.1 ± 0.1 Mg·ha^−1^·year^−1^, respectively [*t* = −4.8, *P* < 0.0001]), especially at end of April 2009 (Fig. 2A). Likewise, flux of flowers plus fruits of *C. elastica* was twice as fast as that of native species (Table 2), with a peak in June 2008 (Fig. 2B). Leaves of *C. elastica* were the most abundant component of total litter fall (38%), followed by the leaves of native species (32%), flowers plus fruits of *C. elastica* (6%), and of native species (2%). Annual litter fall for these stands was 9.6 ± 0.5 Mg·ha^−1^·year^−1^, peaking between April and July (F = 8.1, df = 24, *P* < 0.0001).

**Table 2:** Total annual litter fall, mean litter standing stock, and litter decomposition for leaves and reproductive components of *Castilla elastica* and of grouped native species. Data shown separately for two stands (1 = Tallonal 1; 2 = Tallonal 2) at novel forests of *C. elastica*. F + F = flowers plus fruits

	Castilla elastica			Native species			
	1	2	Mean	1	2	Mean	All
Annual litter fall (g·m^−2^ year^−1^)							
Leaves	255.5	511	383.2	182.5	109.5	146	529.2
F + F	182.5	73	127.7	73	25.5	49.2	177
Total	438	584	511	255.5	135	195.2	706.2
Mean litter standing stock (g·m^−2^)							
Leaves	59	119	89.8	108	29	69.08	158.9
F + F	3.4	5.3	4.3	20	1.3	10.6	15.05
Total	62.4	124.3	93.3	128	32.3	80.1	173.9
Decomposition rates (*k*·year^−1^) and number of days for decomposition							
Leaves	7.02	4.7	5.8	2	4.18	3.09	3.33
Days_Leaves	52	78	65	182	87	134.5	109.6
F + F	53.6	13.7	33.3	3.6	19.6	11.6	11.7
Days_F + F	7	27	17	101	19	60	31.1
Total	7.06	4.6	5.8	1.9	4.17	3.03	4.06
Days_Total	52	79	65.5	192	88	140	89.9

#### Litter standing stock

**Figure 3:**
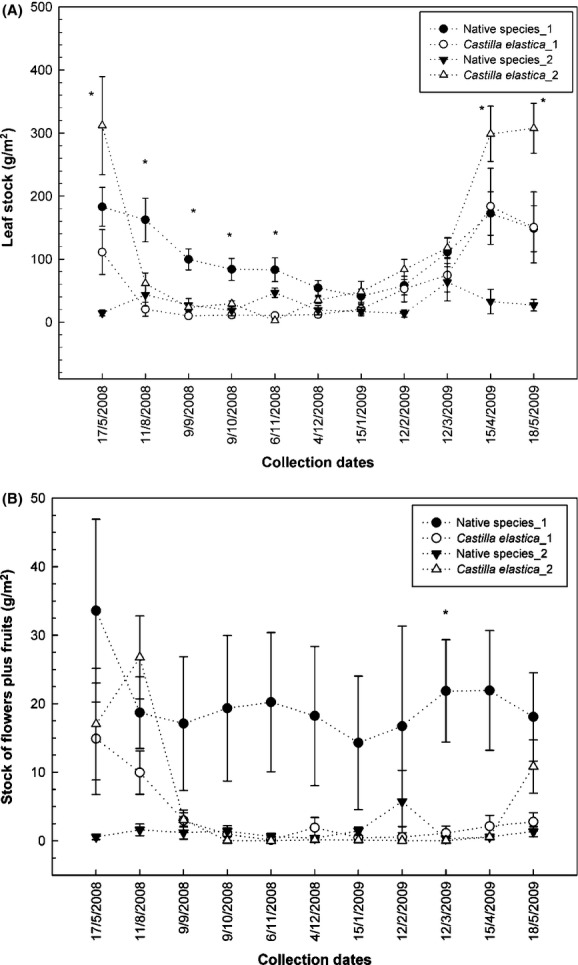
(A) Litter standing stock of leaves of *Castilla elastica* and grouped native species. 1 = Tallonal 1; 2 = Tallonal 2. (B) Stock of flowers plus fruits. Numbers indicate study sites identification. (*) = *P* ≤ 0.05 among species.

Leaf litter stock of *C. elastica* was higher than that of native species (89 ± 9 and 69 ± 5 g·m^−2^, respectively [*t* = −1.9, df = 358, *P* < 0.049]; Table 2). The highest peaks occurred during May 2008 and April/May 2009, after the dry season (F = 31.4, df = 10, *P* < 0.0001 [Fig. 3A]). Instead, stock of flowers plus fruits was lower for *C. elastica* than for native species: 4.3 ± 0.7 and 10.6 ± 1.5 g·m^−2^, respectively (*t* = 3.5, df = 317, *P* < 0.0004; Fig. 3B).

Litter wood mass and miscellaneous fragments remained approximately constant throughout the year (mean ± SE = 183 ± 9 g·m^−2^ and 54 ± 4 g·m^−2^, respectively), but displayed relatively lower mass in April/May 2009 (134 ± 21 g·m^−2^ and 10 ± 1.9 g·m^−2^ for wood and miscellaneous fragments, respectively). The major component of the litter stock, by weight, was wood, followed by leaves of *C. elastica* and leaves of native species (41, 18, and 16 percent, respectively). Mean litter standing stock of the forest was 4.47 ± 0.14 Mg·ha^−1^ with a peak of accumulation in May 2008 (6.6 ± 0.4 Mg·ha^−1^).

#### Litter turnover

Turnover rate of leaves plus flowers plus fruits was 4.06 *k*·year^−1^. However, faster rates were reported for only components of *C. elastica* (5.8 *k*·year^−1^) compared to those of native species (3.09 *k*·year^−1^ [Table 2]). Annual total litter turnover was 2.18 *k*·year^−1^, corresponding to 5.5 months of residence time. The fastest decomposition rate occurred between August and November 2008, and the highest stock was measured in January and from March to May (Appendix 4).

## Discussion

### Survival rates and ecological strategies of juvenile plants

I found that the relative survival rate of *C. elastica* juvenile plants was lower than that of grouped native species: 28 and 53%, respectively. Overall, species at El Tallonal showed two classic general strategies of development in early plant stages: *C. elastica* showed high fecundity and mortality, and native species showed the opposite strategy. In fact, native species usually have scarcer and more persistent juvenile individuals compared to those of introduced species (Maron and Vilà 2001; Daehler 2003). A meta-analysis by Daehler (2003) showed that native species survive better than introduced species in a disturbed habitat. Likewise, Fuentes-Ramírez et al. (2011) reported higher survival rate (70%) for *Cryptocarya alba* (Molina) Looser, a native species, in invaded regenerating stands. The high dominance combined even with low survivorship suggest that propagule abundance is higher for *C. elastica* than for native plants.

According to other comparative studies, naturalized species are likely to perform better than native species during the establishment, showing higher survivorship for example (Canham 1989; Kobe 1997; Sanford et al. 2003). This hypothesis is partially supported by the results presented in this study. The absolute higher abundance *C. elastica*'s seedlings and saplings (96% of total individuals) reinforces the sentence above. Furthermore, *C. elastica's* high dominance suggests a better performance in the early stages of development, as well as give us light to understand the high dominance of *C. elastica* in the subsequent plant stages in these novel forests.

### Tree growth: contrasting trends for species and ecosystems

Native species are often reported as having slower growth rates than introduced species (Pattison et al. 1998; Grotkopp and Rejmánek 2007; Fuentes-Ramírez et al. 2011). Overall, we found that native and naturalized species showed similar tree growth rates when compared to that of *C. elastica*. However all rates were lower than that of *G. guidonia*, which grew at least four times higher than *C. elastica*. It is likely that *G. guidonia* has adjusted rapidly to the new environmental conditions at *Castilla* novel forests or that the high stem growth rate is an intrinsic trait of this species. The fact that most of the species had about the same tree growth rates suggest similar mechanisms of biomass storage in the community regardless of their status as native or introduced.

The stem growth rates found here were in the same range of variation of native trees in moist secondary forests in Puerto Rico: 4.9 ± 2.9 to 40 ± 6.9 cm^2^·year^−1^ (Silver et al. 2004), but slower than those in other tropical regions. For example, large trees (>35 cm DBH) at a mature NSF in Panama grew at rates of 21 cm^2^·year^−1^ (Milton et al. 1994), while the large trees in the present study grew by 12.9 cm^2^·year^−1^.

Because of the influence of introduced species, that usually display fast growth rates, novel forests are expected to have faster stand growth rates than NSFs. Instead, stands of Castilla showed growth rates comparable to NSFs (Table 3). For example, Fearnside and Guimarães (1996) reported a mean stand growth rate of ∼2 Mg·ha^−1^·year^−1^ after 80 years of forest regeneration in a NSF. Thus, mature novel ecosystems as Castilla forest can show not only structural (Aide et al. 1996; Fonseca-da Silva 2014), but also functional traits that are similar to NSFs after some decades of regeneration.

**Table 3:** Attributes of novel forests of *Castilla elastica* and other lowland subtropical and tropical moist* forests. Blank spaces mean no data

Place	Site name (classification)	Soil/substrate	Age (years)	Litter fall (Mg·ha^−1^·year^−1^)	Litter standing stock (Mg·ha^−1^)	Litter turnover (*k*·year^−1^)	Stand-level growth (Mg·ha^−1^·year^−1^)	Source
Puerto Rico	Novel forests of *Castilla elastica*	Oxisol/karst	40–50	9.6	4.4 ± 0.14	2.18; 4.06§	2.0 ± 0.4	Present study
Puerto Rico	Novel forests of *Spathodea campanulata*	karst	38	13.3				Abelleira-Martínez (2011)
Puerto Rico	Novel forests of *Spathodea campanulata*	Alluvial			4 ± 0.9 to 5.5 ± 0.7			Abelleira-Martínez and Lugo (2008)
Puerto Rico	Native secondary forest	Oxisol/volcanic	70	11.7	7.5 ± 0.05	1.09 ± 0.1		Ostertag et al. (2003)
Puerto Rico	Native secondary forest	karst	40	8.4	2.9	0.8		Ruiz-Jaen and Aide (2005)
Borneo	Previous logged dipterocarp forests		19				2.7 ± 2.3	Berry et al. (2010)
Brazil	Native secondary forest		14–19	5.4–13.4				Barlow et al. (2007)
Brazil	Abandoned pasture (Altamira) (NSF)		20				3.4	Fearnside and Guimarães (1996)
Colombia	Evergreen seasonal forest (NSF)		>25	9.5				Folster and de las Salas (1976)
Ghana	Semi-deciduous forest (NSF)		>25	10.7				Nye (1961)
Guatemala	Native secondary forest		∼50	9				Ewel (1976)
Mexico	Abandoned cornfield (NSF)	Andosols/volcanic	50		6.8 ± 0.6		5.7	Flint-Hughes et al. (1999)
Nigeria	Native secondary forests		>25	7.2^L^				Hopkins (1966)
Puerto Rico	Reforested forests (NSF)	Ultisols/volcanic	55	10.6 ± 0.5			2.8 ± 0.1	Silver et al. (2004)

§, Flowers plus fruits; L, Leaves necromass; *, classification *sensu* Holdridge (1967).

### Litter dynamics: faster fluxes for Castilla

Leaf fall *C. elastica* displayed twice as much as that of native species (Table 2). The larger litter mass from *C. elastica* (44% of total, including leaves, flowers and fruits) circulating in the ecosystem suggests a strong effect of this species on the aboveground dynamics of the novel forest. In this case, about half of the biomass that is present in the nutrient cycling, decomposition, carbon storage, and other fluxes is released by only one species. Thus, the management of *C. elastica* trees in these novel forests has to be carefully considered, because changes to the population density are likely to reflect modifications to the function of the ecosystem.

*Castilla elastica* leaves showed a higher biomass turnover rate than the leaves of native species (5.8 vs 3.09 *k*·year^−1^ [Table 2]), suggesting that these leaves are more labile than those of native species. Perhaps, factors such as lower C/N ratios or higher nutrient content, features are typically found in introduced species, are predominant in these novel forests (Baruch and Goldstein 1999; Ehrenfeld 2003). As a consequence of those functional traits, introduced species could accelerate the nutrient cycling of the ecosystem by delivering high-quality litter fall (Ashton et al. 2005).

Novel forests of *C. elastica* had total litter fall in the range of variation for NSFs and NFs on karst soils in Puerto Rico: 9.6 vs. 8.4 to 13.3 Mg·ha^−1^·year^−1^ (Ruiz-Jaén and Aide 2005; Abelleira-Martínez 2011). Such rates are also similar to those in regenerating NSFs in other substrates and of similar age (Zou et al. 1995).

Litter standing stock was lower in *Castilla* novel forests than in NSFs around the Tropics: 4.4 ± 0.14 vs. 4.9 to 7.5 Mg·ha^−1^, respectively (Table 3). Relative to other secondary forests in Puerto Rico, the litter standing stock in *Castilla* NFs was approximately 25–35% lower than NSFs. For example, a wet forest in Puerto Rico of about 30 years old showed 5.8 ± 0.1 Mg·ha^−1^ of litter standing stock (Li et al. 2005). Apparently, differences in the relative amounts of litter components have changed the overall rates of processes in NFs of *Castilla*, which has mostly been driven by the high amount of labile *C. elastica's* biomass. The Olson decomposition constant (*k*) for Castilla NFs was high throughout the year, showing twice as fast decomposition than what is expected for secondary forests in the moist tropics. These data support the notion that dominant introduced species can change forest functioning, in this case, leading to faster litter fluxes than native ecosystems.

Native secondary forests are known to be important in terms of biomass storage (Brown and Lugo 1990). Additionally, Mascaro et al. (2012) reported that carbon fluxes in novel ecosystems are likely to be high. Here, I have shown empirical supporting evidence for both statements. Attributes reported for *C. elastica* novel forests show high potential for biomass accumulation in the vegetation, as well as fast cycling of aboveground biomass.

## Conclusions and management implications

In the present study, I demonstrate differences in the ecology of introduced and native species, as well as between attributes of novel and native secondary forests. The dominant introduced species, *C. elastica*, has lower survival rates and probably different life growth strategy than native species found as seedlings and saplings in the same community. Adult trees of a native species, *G. guidonia*, have faster growth rates compared to those of the *C. elastica* and to the other species. Novel forests of *C. elastica* show similar stand biomass growth and lower litter standing stock on average than NSFs. Turnover of ground litter is faster in NFs of Castilla, especially for fine leaf necromass of *C. elastica*. In general, results demonstrate that *C. elastica* novel forests are comparable or even faster in some functional attributes than NSFs.

Although the juvenile survivorship was lower for *C. elastica*, its population constitutes the majority of individuals during all stages of development. Nonetheless, *Castilla* NFs have offered favorable conditions for the co-development of native species. The present ecological state of this novel ecosystem shows dynamic functioning and probably valuable ecological services associated with the fast biomass fluxes. In fact, all the above highlight the importance of *C. elastica* NFs for conservation.

Conservation guidelines for novel ecosystems are needed to preserve the valuable ecological services they provide, such as the maintenance of hydrology and carbon services, as well as the mitigation for climate change (Hobbs et al. 2013). Further studies focused on the ecology, resilience, and stability of emerging ecosystems are core for understanding their dynamics at larger spatial and temporal scales and to establish suitable management practices. Ignoring the presence of novel ecosystems or their abundance on a global scale as well as adopting unsuitable management decisions may be harmful to the biodiversity contained in these forests.

## Appendix 1

Temperature (A), relative humidity (B), photosynthetic active radiation—PAR (C), and soil water content (D) of novel forests of *Castilla elastica* for the period of study.

## Appendix 2: List of taxa and relative frequency of juvenile plants at *Castilla* novel forests. Na = native, In = introduced naturalized

**Table:**

Taxa	Relative frequency (%)
*Castilla elastica*^In^	96.3
*Guarea guidonia*^Na^	1.3
Leguminosae	0.3
*Piper aduncum*^Na^	0.3
*Syzygium jambos*^In^	0.3
*Eugenia biflora*^Na^	0.2
Unknown 1	0.1
*Casearia guianensis*^Na^	0.1
*Chrysophyllum argenteum*^Na^	0.1
*Coffea arabica*^In^	0.1
*Cordia alliodora*^Na^	0.1
*Cupania americana*^Na^	0.1
*Faramea occidentalis*^Na^	0.1
*Guapira fragans*^Na^	0.1
*Inga vera*^Na^	0.1
*Ocotea coriacea*^Na^	0.1
*Ocotea leucoxylon*^Na^	0.1
Piper sp.	0.1
Rubiaceae	0.1
*Trichilia pallida*^Na^	0.1
Unknown 2	0.1
Unknown 3	0.1
Unknown 4	0.1

## Appendix 3: Weighed stand wood density, tree height, and density of novel forests of *Castilla elastica*. Large trees (L) = ≥10 cm of diameter at breast height (DBH), small trees (S) = ≥2.5 < 10 cm of DBH, and all trees (A) = ≥2.5 cm of DBH

**Table:**

	Stand wood density* (g·cm^−3^)	Stand tree height (m)			Tree density* (stems·ha^−1^)		
Site	A	L	S	A	L	S	A
Tallonal 1	0.37	18.5	6.4	11.0	608	615	1223
Tallonal 2	0.29	20.0	6.1	11.3	439	444	883
Mean	0.33	19.0	6.2	12.5	513	526	1039

*Fonseca-da Silva (2014).

## Data Accessibility

All data used in this manuscript are present in the text, appendices, and tables.
